# Supplementary figures and images for: Characterisation of cell-scale signalling by the core planar polarity pathway during Drosophila wing development
Source: eLife. 2025 Dec 5;14:RP107947. doi: 10.7554/eLife.107947 (PMC12680375; doi:10.7554/eLife.107947)

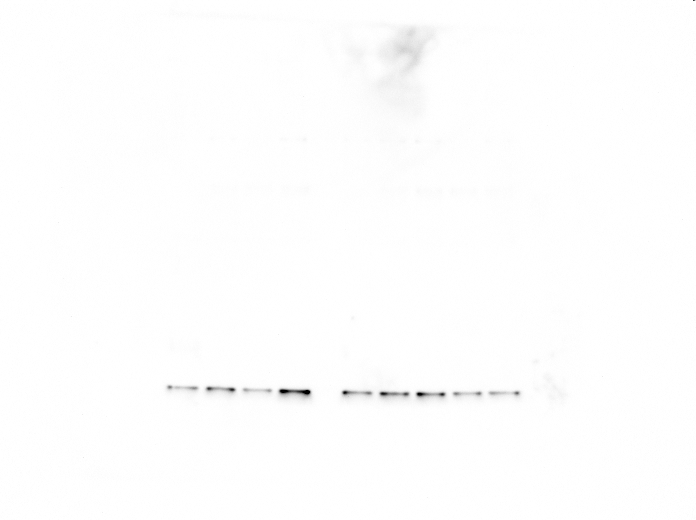

Supplement: Figure 1—figure supplement 1—source data 1. [file elife-107947-fig1-figsupp1-data1.zip › Figure 1-figure supplement 1-source data 1/Fz-mKate2-sfGFP induction Actin.tif]

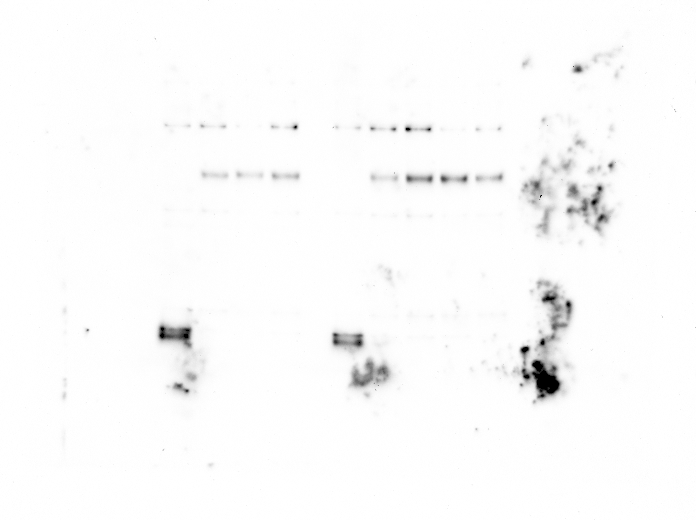

Supplement: Figure 1—figure supplement 1—source data 1. [file elife-107947-fig1-figsupp1-data1.zip › Figure 1-figure supplement 1-source data 1/Fz-mKate2-sfGFP induction Fz.tif]

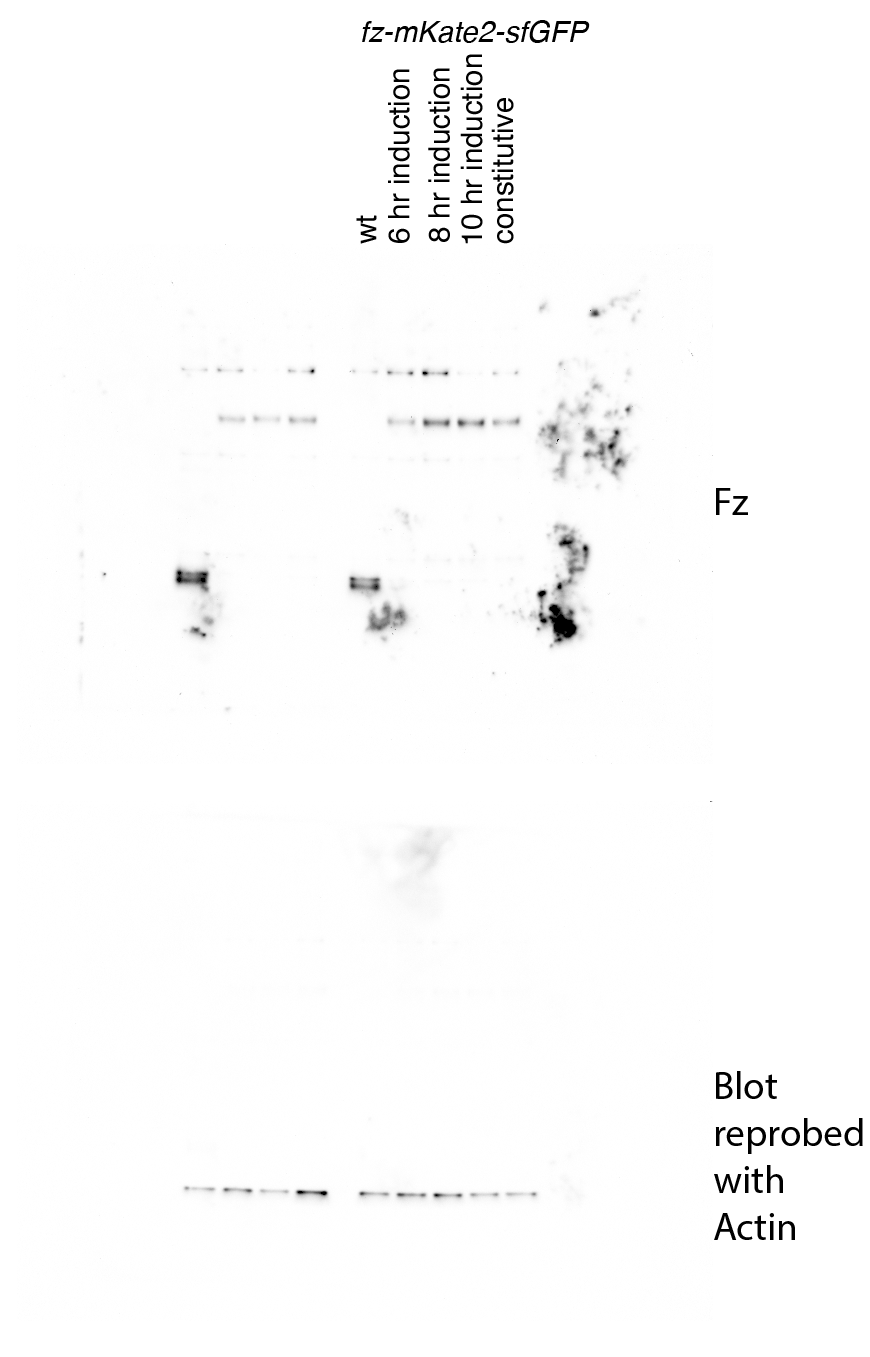

Supplement: Figure 1—figure supplement 1—source data 2. [file elife-107947-fig1-figsupp1-data2.zip › Figure 1-figure supplement 1-source data 2/Fz-mKate2-sfGFP induction Fz-Actin.tif]

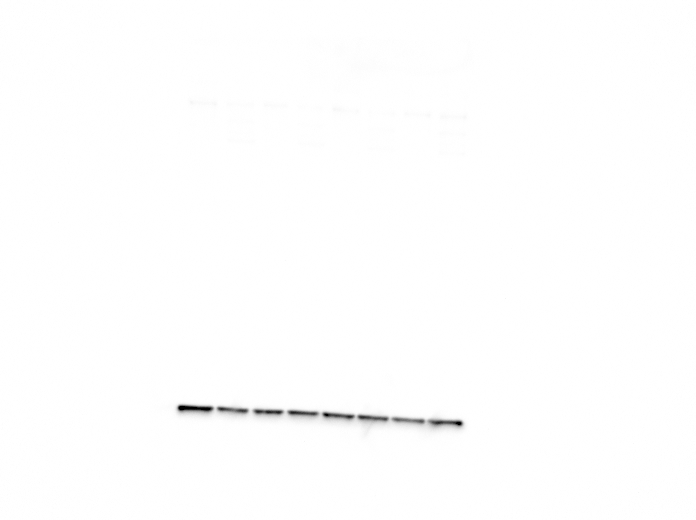

Supplement: Figure 3—figure supplement 1—source data 1. [file elife-107947-fig3-figsupp1-data1.zip › Figure 3-figure supplement 1-source data 2/170125 wt vs fmiE59 het Tub 10sec.tif]

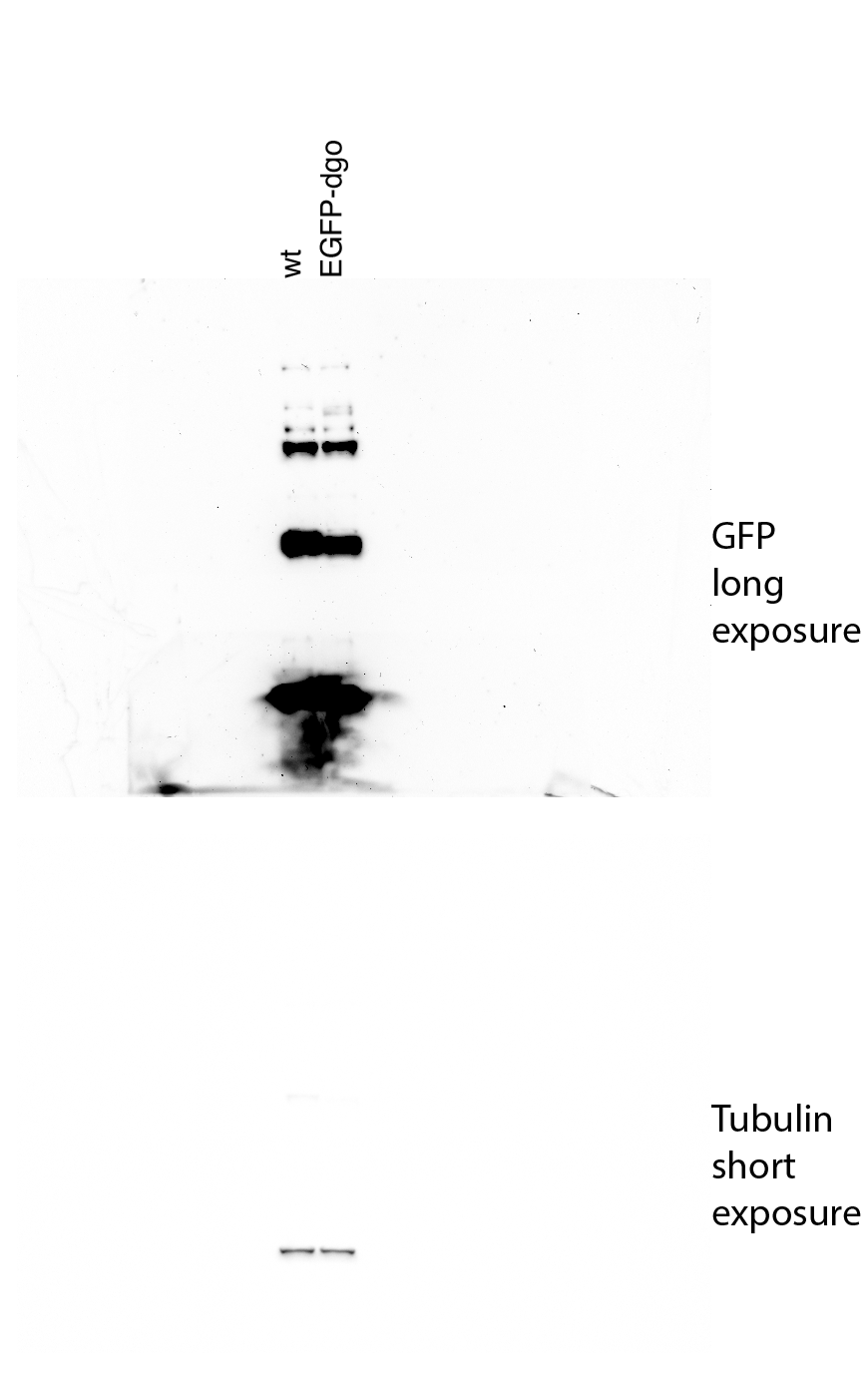

Supplement: Figure 3—figure supplement 1—source data 1. [file elife-107947-fig3-figsupp1-data1.zip › Figure 3-figure supplement 1-source data 2/D EGFP-Dgo vs wt GFP-Tub.tif]

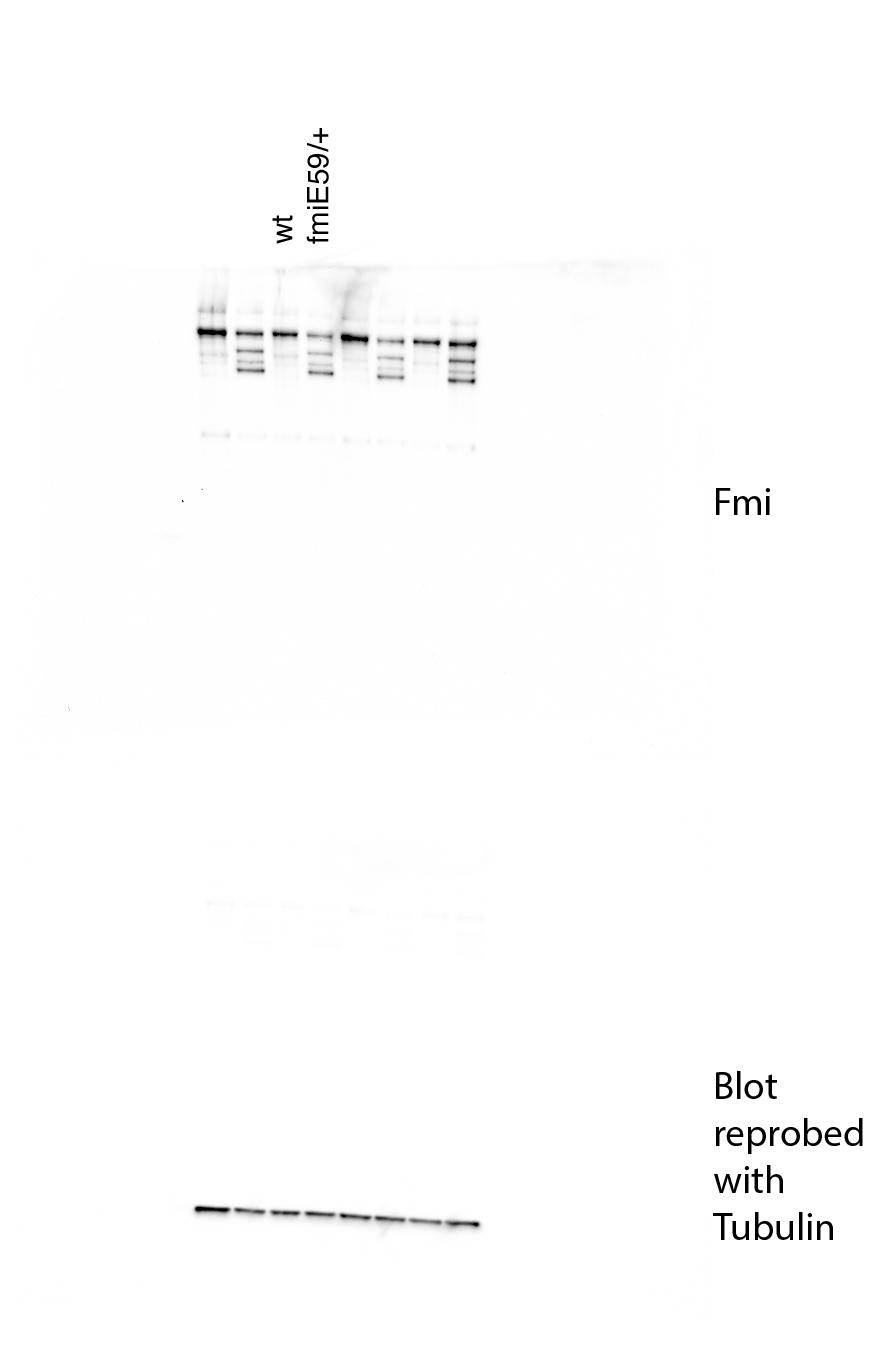

Supplement: Figure 3—figure supplement 1—source data 1. [file elife-107947-fig3-figsupp1-data1.zip › Figure 3-figure supplement 1-source data 2/B wt vs fmiE59 het Fmi-Tub.tif]

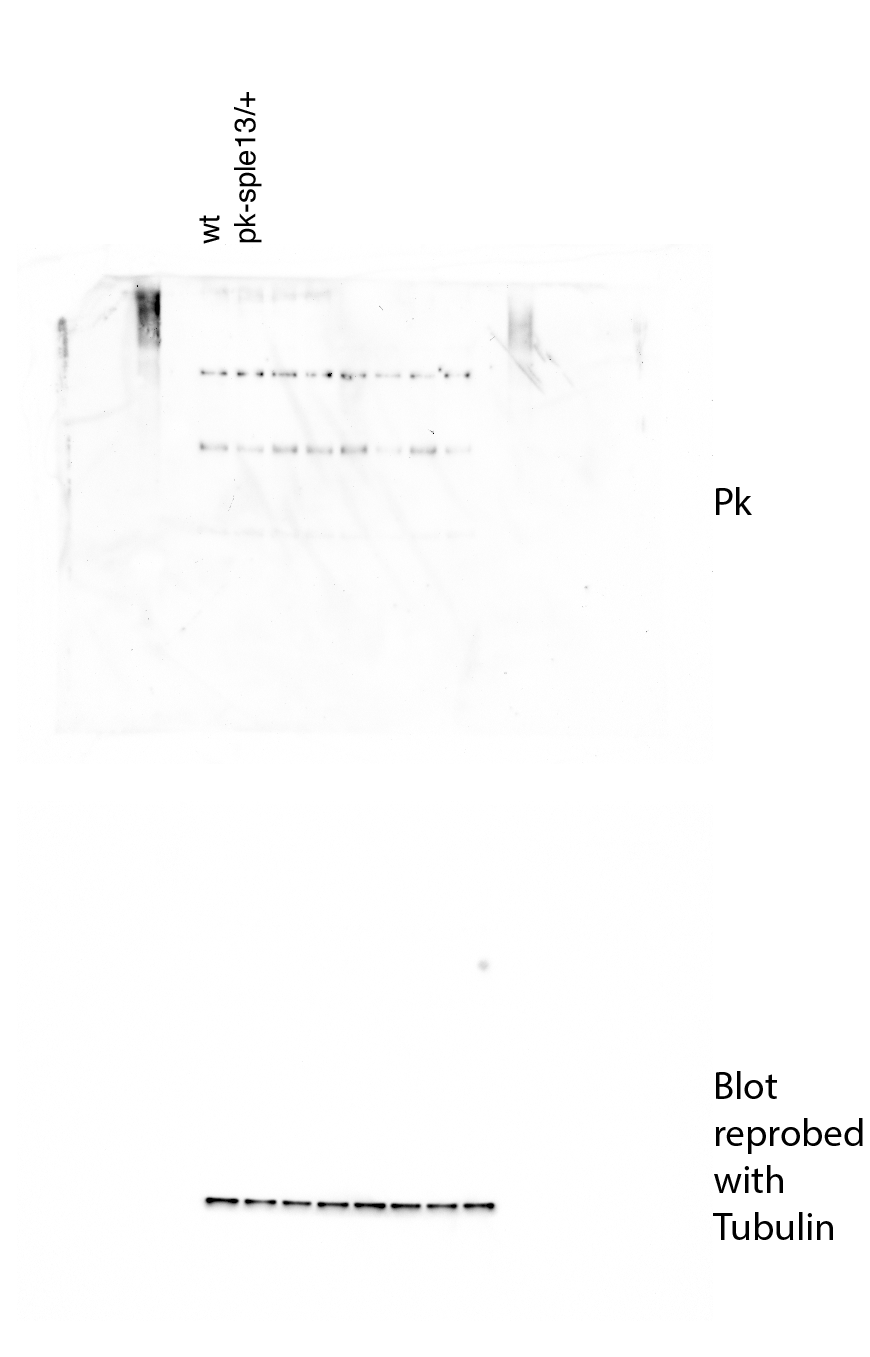

Supplement: Figure 3—figure supplement 1—source data 1. [file elife-107947-fig3-figsupp1-data1.zip › Figure 3-figure supplement 1-source data 2/A wt vs pksple13 het Pk-Tub.tif]

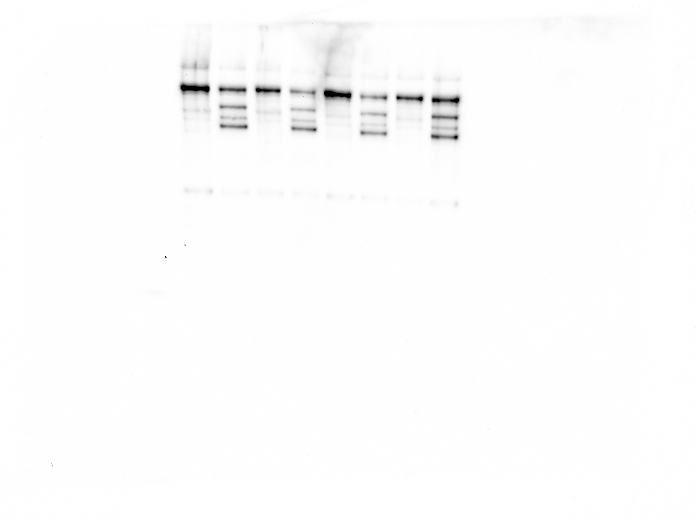

Supplement: Figure 3—figure supplement 1—source data 1. [file elife-107947-fig3-figsupp1-data1.zip › Figure 3-figure supplement 1-source data 2/160125 wt vs fmiE59 het Fmi 5min.tif]

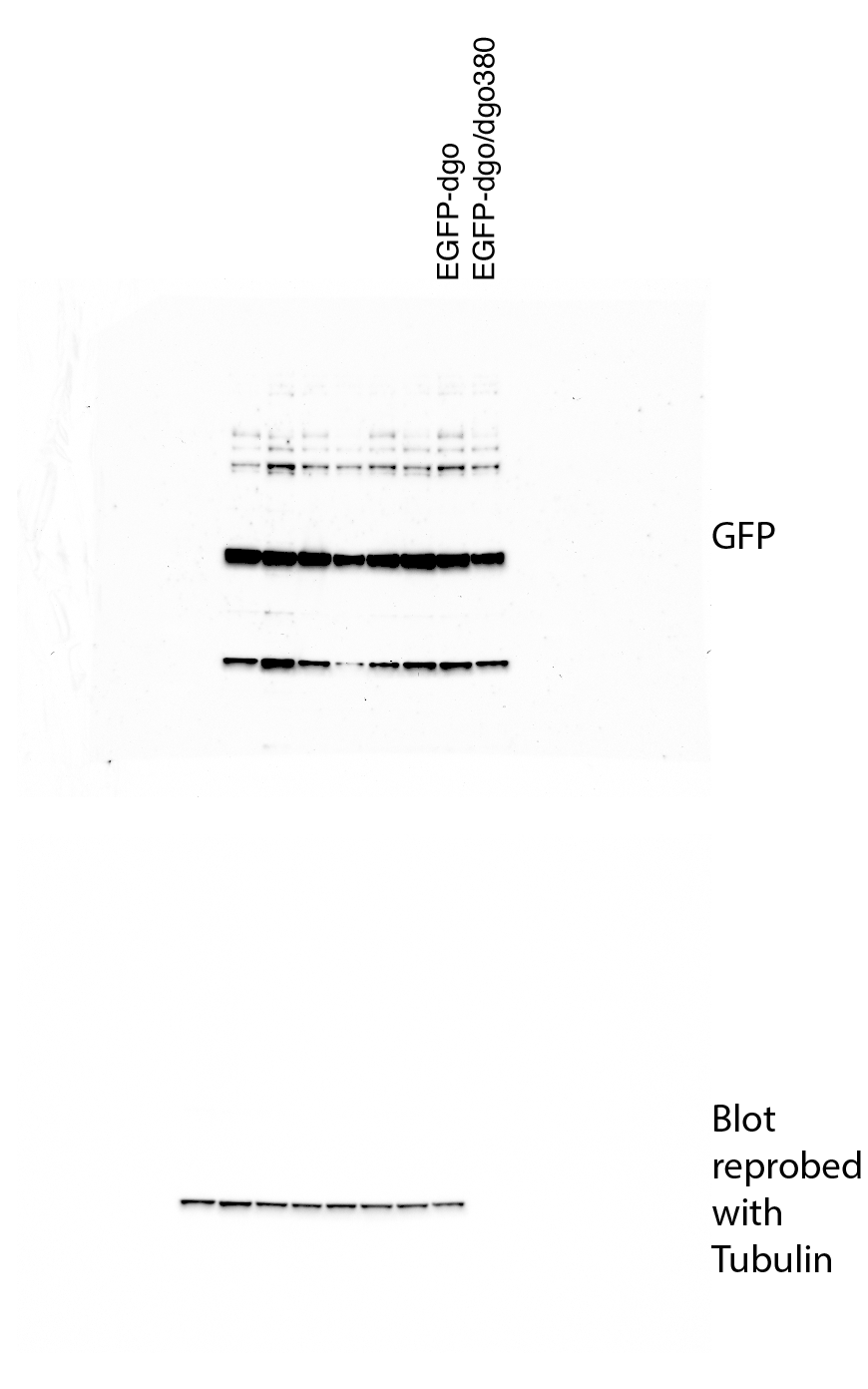

Supplement: Figure 3—figure supplement 1—source data 1. [file elife-107947-fig3-figsupp1-data1.zip › Figure 3-figure supplement 1-source data 2/C EGFP-Dgo vs EGFP-dgo_dgo GFP-Tub.tif]

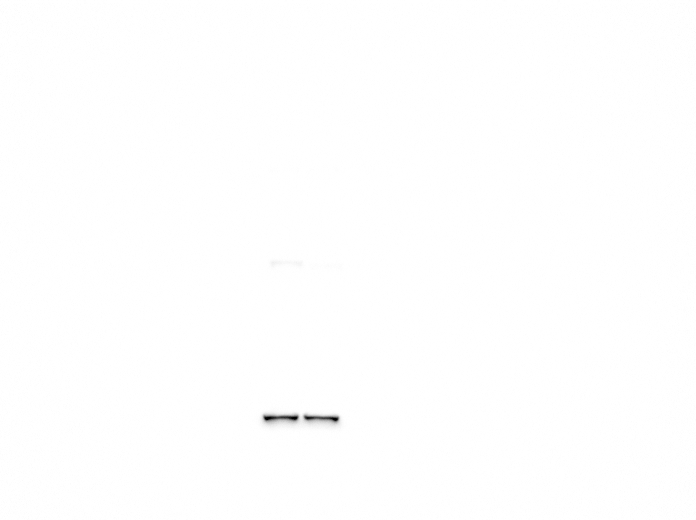

Supplement: Figure 3—figure supplement 1—source data 2. [file elife-107947-fig3-figsupp1-data2.zip › Figure 3-figure supplement 1-source data 1/D EGFP-Dgo vs wt Tub.tif]

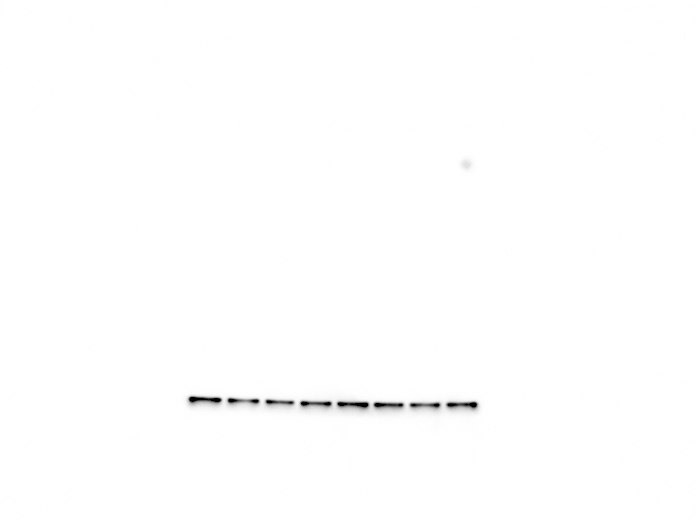

Supplement: Figure 3—figure supplement 1—source data 2. [file elife-107947-fig3-figsupp1-data2.zip › Figure 3-figure supplement 1-source data 1/A wt vs pksple13 het Tub.tif]

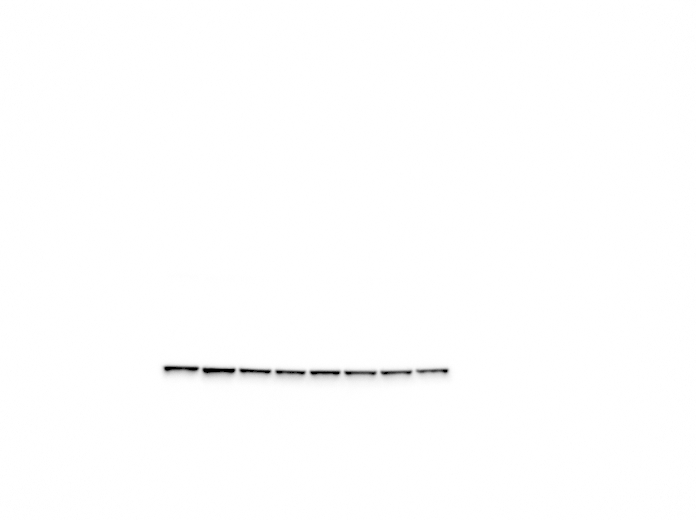

Supplement: Figure 3—figure supplement 1—source data 2. [file elife-107947-fig3-figsupp1-data2.zip › Figure 3-figure supplement 1-source data 1/C EGFP-Dgo vs EGFP-dgo_dgo Tub.tif]

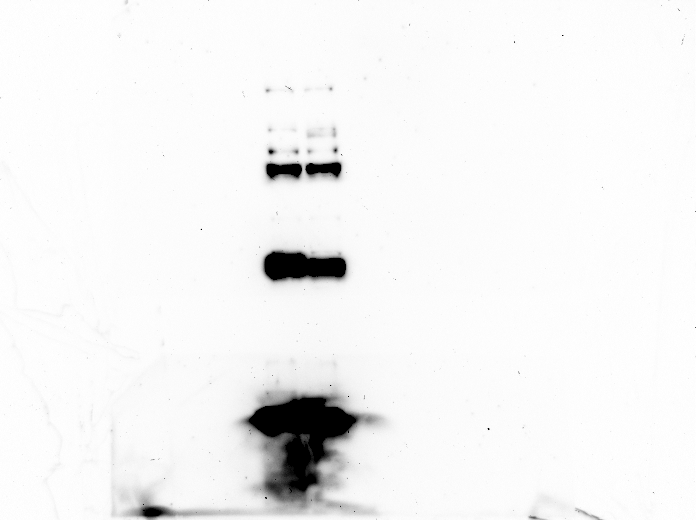

Supplement: Figure 3—figure supplement 1—source data 2. [file elife-107947-fig3-figsupp1-data2.zip › Figure 3-figure supplement 1-source data 1/D EGFP-Dgo vs wt GFP.tif]

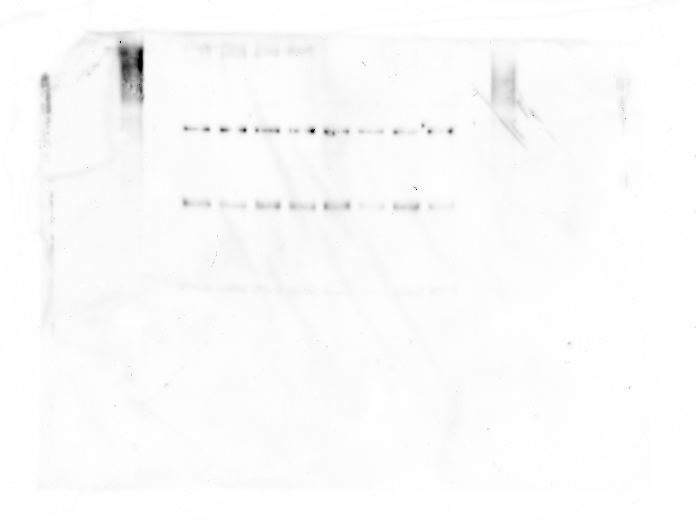

Supplement: Figure 3—figure supplement 1—source data 2. [file elife-107947-fig3-figsupp1-data2.zip › Figure 3-figure supplement 1-source data 1/A wt vs pksple13 het Pk.tif]

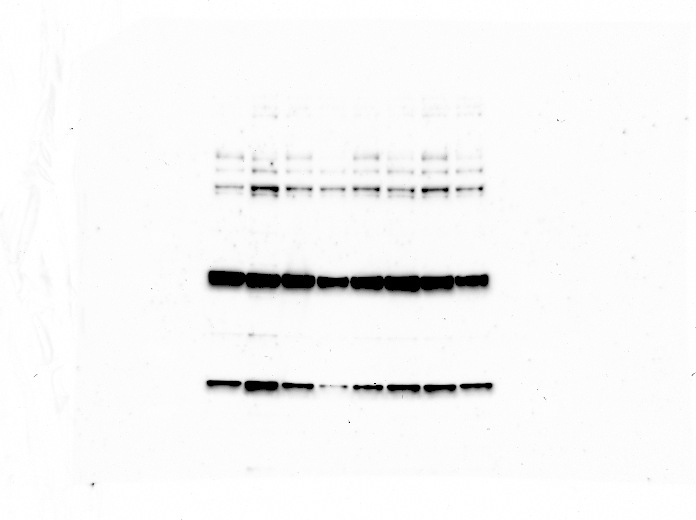

Supplement: Figure 3—figure supplement 1—source data 2. [file elife-107947-fig3-figsupp1-data2.zip › Figure 3-figure supplement 1-source data 1/C EGFP-Dgo vs EGFP-dgo_dgo GFP.tif]
