# Supplementary material for: Characterisation of cell-scale signalling by the core planar polarity pathway during Drosophila wing development
Source: eLife. 2025 Dec 5;14:RP107947. doi: 10.7554/eLife.107947 (PMC12680375; doi:10.7554/eLife.107947)
Supplement: Supplementary file 2. — PDF file containing new Matlab scripts used for data analysis. [file elife-107947-supp2.docx]

**Supplemental Materials – Analysis script 1**

**Combined results for polarity (magnitude + angle)**

This is a MATLAB script suitable for combining polarity magnitude and angle results generated by QuantifyPolarity software.

This script is designed to analyse files for 4 conditions (row1, row2, row3 and row4). In order to use it you have to strictly follow the following architecture:

- Create a folder, for row1 analysis, named ‘Analysed_sfGFP_row1’ containing analysed data from QuantifyPolarity software which own ‘Result’ subfolder. Do the same thing for row2 up to row4.
- Run the script and choose the appropriate folder location when prompted.

The script redistributes polarity angles in a range between -45° and 135° from −90° and +90° for each analysed cells per wing for all conditions. For each wing of the same condition (for example ‘row1’), those reoriented angles are averaged as polarity magnitude and added in a new saved .csv file ‘Analysed_sfGFP_row1’ in column ‘Average_Reoriented_Angle_Polarity_deg’. For each condition, the mean of polarity magnitude and angle is calculated and added in a saved new .csv file ‘Average_Reoriented_Angle_Polarity_row1’.

clc

clear all

close all

warning off

%% To locate file

currdir = pwd;

addpath(pwd);

filedir = uigetdir();

cd(filedir);

rootdir=filedir;

myfolder = [filedir];% location of the folder with files to convert

%% Get a list of all csv files in the current folder, or subfolders of it.

filePattern = fullfile(myfolder, '\Analysed*', '\*', '\Result', 'PCA_Cell-by-Cell_Polarity.csv'); % Change to whatever pattern you need.

fds = fileDatastore(filePattern, 'ReadFcn', @importdata);

fullFileNames = fds.Files;

numFiles = length(fullFileNames);

% Loop over all files reading them in and plotting them.

for k = 1 : numFiles

% fprintf('Now reading file %s\n', fullFileNames{k});

% Now have code to read in the data using whatever function you want

A = readtable(fullFileNames{k});

A.Properties.VariableNames = ["Cell Identity", "PCA Magnitude", "PCA Angle(degrees)"];

%% To redistribute polarity angles in a range between -45° and 135° from −90° and 90° for each analysed cells per wing

B = xlsread(fullFileNames{k},'C:C'); % to transform data in column C

x = B;

y = numel(x);

for i = 1:y

if x(i)<=90 && x(i)>=90

elseif x(i)<-45

z(i)=180+x(i);

elseif x(i)>-45

z(i)=x(i);

end

end

B=array2table(z(:)); % this converts the results into a table format.

B.Properties.VariableNames = ["Reoriented PCA Angle(degrees)"];

C = [A, B];

writetable(C, fullFileNames{k});

clearvars z;

end

%% Get a list of all csv files in the current folder, or subfolders of it.

filePattern = fullfile(myfolder,'\Analysed*', '\*', '\Result', 'PCA_Cell-by-Cell_Polarity.csv'); % Change to whatever pattern you need.

filePattern1 = fullfile(myfolder,'\Analysed*', '\*', '\Result');

fds1 = fileDatastore(filePattern1, 'ReadFcn', @importdata);

fullFileNames1 = fds1.Files;

filePattern2 = fullfile(myfolder,'\Analysed*', '\*', '\Result', '*PCA_Average_Polarity_Magnitude.csv');

fds = fileDatastore(filePattern, 'ReadFcn', @importdata);

fullFileNames = fds.Files;

fds2 = fileDatastore(filePattern2, 'ReadFcn', @importdata);

fullFileNames2 = fds2.Files;

filePattern3 = fullfile(myfolder,'\Analysed*', '\*', '\Result', '*PCA_Average_Polarity_Magnitude.csv');

fds3 = fileDatastore(filePattern3, 'ReadFcn', @importdata);

fullFileName3 = fds3.Files;

numFiles = length(fullFileNames);

% Loop over all files reading them in and plotting them.

for k = 1 : numFiles

A = readtable(fullFileNames{k});

D = readtable(fullFileNames2{k});

%% To get Average of Polarity angle

B = xlsread(fullFileNames{k},'D:D'); %to transform data in column D

data = B;

x = data;

y = numel(x);

ang_rad = deg2rad (x);

x(x == 0) = NaN;

data_avg = sum(exp(1i*ang_rad(:)));

% obtain mean by

mu = angle(data_avg);

Average_Polarity_Angle_mean_degrees = rad2deg(mu);

T = array2table(Average_Polarity_Angle_mean_degrees);

T.Properties.VariableNames(1:1) = {'Average_Reoriented_Angle_Polarity(deg)'};

Reoriented_PCA_Average_Polarity_Magnitude = [D T]; % combine '*_PCA_Average_Polarity_Magnitude' with 'Average_Reoriented_Angle_Polarity'

R = Reoriented_PCA_Average_Polarity_Magnitude;

writetable(R,['Reoriented_PCA_Average_Polarity_Magnitude_',num2str(k),'.xlsx']);

filePattern4 = fullfile(myfolder, '*.xlsx' ); % Change to whatever pattern you need.

% fileDatastore(location,"ReadFcn",@customreader,"FileExtensions",[".exts",".extx"]).

fds4 = fileDatastore(filePattern4, 'ReadFcn', @importdata);

fullFileNames4 = fds4.Files;

numFiles4 = length(fullFileNames4);

% Loop over all files reading them in and plotting them.

Folder1 = filePattern1; % asks user for a folder

FilePattern = fullfile(Folder1,'*PCA_Average_Polarity_Magnitude*.csv');

Files1 = dir(FilePattern); % puts files into struct array

for n = 1 : 1 : numFiles4

% Get input filename.

fullFileName = fullfile(Files1.folder, Files1.name);

% fprintf('Reading "%s".\n', fullFileName);

% Call your function to read the .dta file and put it into a table variable.

properFolder2 = myfolder;

properFilePattern = [myfolder, '\Reoriented_PCA_Average_Polarity_Magnitude_*'];

properFiles2 = dir(properFilePattern); % puts files into struct array

properfullFileName = fullfile(properFiles2.folder, properFiles2.name);

properthisTable = readtable(properfullFileName);

thisTable = readtable(properfullFileName);

R_Trace_data{n} = properthisTable;

% Get output filename. It's the same except the extension is txt instead of dta.

[filepath,name,ext] = fileparts(fullFileName);

outputFullFileName = [name '.xlsx'];

% Get input filename.

fullFileName5 = fullfile(Files1(k).folder, Files1(k).name);

outputFullFileName = strrep(fullFileName5, '.xlsx', '.csv');

writetable(R_Trace_data{n}, outputFullFileName);

delete (myfolder, '*Reoriented*');

end

end

delete (myfolder, '*Reoriented*');

%% Analysis row1, to combine wings results in 1 .csv file

rootdir=[filedir,'\Analysed_sfGFP_row1']; % ‘Analysed_sfGFP_row1’ is the name used there to put

myFiles = (fullfile(rootdir,'\Analysed_sfGFP_row1', '\*', '\Result', '*PCA_Average_Polarity_Magnitude.csv')); % Get list of .csv files to combine (with this script, the file with PCA_Average_Polarity_Magnitude results)

ds = tabularTextDatastore((fullfile(rootdir, '\*', '\Result', '*PCA_Average_Polarity_Magnitude.csv')));

T = readall(ds);

% To name the combined csv file as the folder containing it

file = rootdir;

[filepath,name,ext] = fileparts(file);

filename1 = name;

writetable(T, filename1); % to write the table T, here .txt

% To name the .csv as the folder containing it

dinfo = dir('*.txt');

filenames = {dinfo.name};

for K = 1 : length(filenames)

thisfile = filenames{K};

[basedir, basename, ~] = fileparts(thisfile);

newfile = fullfile(basedir, [basename '.csv']);

writetable(T, newfile)

end

delete('*.txt');

%% Analysis row2, to combine wings results in 1 .csv file

rootdir=[filedir,'\Analysed_sfGFP_row2'];

myFiles = (fullfile(rootdir,'\Analysed_sfGFP_row2', '\*', '\Result', '*PCA_Average_Polarity_Magnitude.csv')); % Get list of .csv files to combine (with this script, the file with PCA_Average_Polarity_Magnitude results)

ds = tabularTextDatastore((fullfile(rootdir, '\*', '\Result', '*PCA_Average_Polarity_Magnitude.csv')));

T = readall(ds);

% To name the combined csv file as the folder containing it

file = rootdir;

[filepath,name,ext] = fileparts(file);

filename1 = name;

writetable(T, filename1); % to write the table T, here .txt

% To name the .csv as the folder containing it

dinfo = dir('*.txt');

filenames = {dinfo.name};

for K = 1 : length(filenames)

thisfile = filenames{K};

[basedir, basename, ~] = fileparts(thisfile);

newfile = fullfile(basedir, [basename '.csv']);

writetable(T, newfile)

end

delete('*.txt');

%% Analysis row3, to combine wings results in 1 .csv file

rootdir=[filedir,'\Analysed_sfGFP_row3'];

myFiles = (fullfile(rootdir,'\Analysed_sfGFP_row3', '\*', '\Result', '*PCA_Average_Polarity_Magnitude.csv')); % Get list of .csv files to combine (with this script, the file with PCA_Average_Polarity_Magnitude results)

ds = tabularTextDatastore((fullfile(rootdir, '\*', '\Result', '*PCA_Average_Polarity_Magnitude.csv')));

T = readall(ds);

% To name the combined csv file as the folder containing it

file = rootdir;

[filepath,name,ext] = fileparts(file);

filename1 = name;

writetable(T, filename1); % to write the table T, here .txt

% To name the .csv as the folder containing it

dinfo = dir('*.txt');

filenames = {dinfo.name};

for K = 1 : length(filenames)

thisfile = filenames{K};

[basedir, basename, ~] = fileparts(thisfile);

newfile = fullfile(basedir, [basename '.csv']);

writetable(T, newfile)

end

delete('*.txt');

%% Analysis row4, to combine wings results in 1 .csv file

rootdir=[filedir,'\Analysed_sfGFP_row4'];

myFiles = (fullfile(rootdir,'\Analysed_sfGFP_row4', '\*', '\Result', '*PCA_Average_Polarity_Magnitude.csv')); % Get list of .csv files to combine (with this script, the file with PCA_Average_Polarity_Magnitude results)

ds = tabularTextDatastore((fullfile(rootdir, '\*', '\Result', '*PCA_Average_Polarity_Magnitude.csv')));

T = readall(ds);

% To name the combined csv file as the folder containing it

file = rootdir;

[filepath,name,ext] = fileparts(file);

filename1 = name;

writetable(T, filename1); % to write the table T, here .txt

% To name the .csv as the folder containing it

dinfo = dir('*.txt');

filenames = {dinfo.name};

for K = 1 : length(filenames)

thisfile = filenames{K};

[basedir, basename, ~] = fileparts(thisfile);

newfile = fullfile(basedir, [basename '.csv']);

writetable(T, newfile)

end

delete('*.txt');

%% To get averaged data by row

myfolder = [filedir];% location of the folder with files to convert

%% Get a list of all csv files in the current folder, or subfolders of it.

filePattern = fullfile(myfolder, 'Analysed_sfGFP_row*.csv'); % Change to whatever pattern you need.

filePattern1 = fullfile(myfolder);

filePattern2 = fullfile(myfolder, 'Analysed_sfGFP_row*.csv');

fds = fileDatastore(filePattern, 'ReadFcn', @importdata);

fullFileNames = fds.Files;

fds2 = fileDatastore(filePattern2, 'ReadFcn', @importdata);

fullFileNames2 = fds2.Files;

numFiles = length(fullFileNames);

% Loop over all files reading them in and plotting them.

for k = 1 : numFiles

A = readtable(fullFileNames{k});

D = readtable(fullFileNames2{k});

% To get Average of Polarity angle

B = xlsread(fullFileNames{k},'J:J'); %to transform data in column D

data = B;

x = data;

y = numel(x);

ang_rad = deg2rad (x);

x(x == 0) = NaN;

data_avg = sum(exp(1i*ang_rad(:)));

% obtain mean by

mu = angle(data_avg);

Average_Polarity_Angle_mean_degrees = rad2deg(mu);

data_mean = Average_Polarity_Angle_mean_degrees;

T = array2table(Average_Polarity_Angle_mean_degrees);

T.Properties.VariableNames(1:1) = {'Average_Reoriented_Angle_Polarity_deg'};

str = 'Average_Reoriented_Angle_Polarity';

t = 0;

t = t + 1;

% To get Average Polarity Magnitude

E = xlsread(fullFileNames{k},'E:E'); %to transform data in column D

data = E;

x = data;

y = numel(x);

% obtain mean by

mu_Polarity = mean (data);

Average_Polarity_Magnitude = mu_Polarity;

data_mean = Average_Polarity_Magnitude;

P = array2table(Average_Polarity_Magnitude);

P.Properties.VariableNames(1:1) = {'Average_Polarity_Magnitude'};

str = 'Average_Polarity_Magnitude';

t = 0;

t = t + 1;

R = [P, T];

writetable(R,['Average_Reoriented_Angle_Polarity_row',num2str(k),'.csv']);

% clc

% clear variables

% close all

fprintf(1, 'Et voilà!');

**Supplemental Materials – Analysis script 2**

**Polar plot for polarity (magnitude + angle)**

This is a MATLAB script suitable for generating polar plot showing polarity magnitude and angle, with results from ‘Combined results for polarity (magnitude + angle)’ MatLab script.

This script draw polar plot, with polarity magnitude and angle for each wing (empty circles) for one condition (example with row1) with results from ‘Analysed_sfGFP_row1’ that is file with averaged data per wing. It is added average polarity magnitude and angle for all wings from the same condition (filled circle) with results coming from ‘Average_Reoriented_Angle_Polarity_row1)

- Open the script, choose which condition to analyse by changing files name ‘'Analysed_sfGFP_row1.csv'’ and ‘'Average_Reoriented_Angle_Polarity_row1.csv'’ by proper files name.
- Run the script.
- Figure is saved in .pdf file with customised title.
- Circle colour and polar plot figure format can by change in the script following instructions.

clc

clear all

close all

warning off

%% To add individual wing results to the graph

myDir = uigetdir ();

cd(myDir);

myFiles = dir(fullfile(myDir, 'Analysed_sfGFP_row1.csv')); %to choose which row to analyse

L = length (myFiles);

for i=1:L

data{i} = readtable (myFiles(i).name);

T = data{i};

PCAMagnitude {i} = T. AveragePolarityMagnitude;

PolarityAngle {i} = T. Average_Reoriented_Angle_Polarity_deg_;

ang_deg = [PolarityAngle {i}]; % angles

ang_rad {i} = ang_deg {i} *pi/180; % convert degrees to radians for polarplot

magnitude {i} = PCAMagnitude {i}; % magnitude to plot

C = ang_rad {i};

D = magnitude {i};

% newcolors = {'#0072BD', '#D95319', '#EDB120', '#77AC30', '#7E2F8E', '#FF0000','#FFFF00', '#FF00FF', '#C0C0C0', '#00FFFF' }; % to choose colors (https://htmlcolorcodes.com/fr/), here set up for 12 different colors, if more than 12 samples, add more colors

newcolors = {'#0072BD'}; % to choose the color

colororder(newcolors)

% s.SizeData = 10;

hs {i} = polarscatter (C, D, 5);

hold on

end

Legend=cell(i,1);

for iter=1:i

Legend{iter}=strcat('row#', num2str(iter));

end

hold on

%% To add averaged results from all wings to the graph

myFiles = dir(fullfile(myDir, 'Average_Reoriented_Angle_Polarity_row1.csv')); %to choose which row to analyse

L = length (myFiles);

for i=1:L

data{i} = readtable (myFiles(i).name);

T = data{i};

PCAMagnitude_Avg {i} = T. Average_Polarity_Magnitude;

PolarityAngle_Avg {i} = T. Average_Reoriented_Angle_Polarity_deg;

ang_deg_Avg = [PolarityAngle_Avg {i}]; % angles

ang_rad_Avg {i} = ang_deg_Avg {i} *pi/180; % convert degrees to radians for polarplot

magnitude_Avg {i} = PCAMagnitude_Avg {i}; % magnitude to plot

G = ang_rad_Avg {i};

H = magnitude_Avg {i};

% newcolors = {'#D95319', '#EDB120', '#EDB120', '#77AC30', '#0072BD', '#D95319', '#EDB120', '#77AC30', '#7E2F8E', '#FF0000','#FFFF00', '#FF00FF', '#C0C0C0', '#00FFFF' }; % to choose colors (https://htmlcolorcodes.com/fr/), here set up for 12 different colors, if more than 12 samples, add more colors

newcolors = {'#0072BD'}; % to choose the color

colororder(newcolors)

hs {i} = polarscatter (G, H, "filled");

hold on

end

Legend=cell(i,1);

for iter=1:i

Legend{iter}=strcat('row#', num2str(iter));

end

%legend(Legend) %to add legend

rlim([0 0.4]); % x-axis scale

thetaticks(0:45:315); % axis scale

figure = hs {i};

% ask title name

answer1 = inputdlg('Enter scatterplot title:'); % ask to get

R1= answer1;

% title_name (R1) % legend

% title_name = R1;

title_name = 'Scatter plot magnitude & angle polarity for sfGFP'; % figure title, avoid special characters, in other case no saved

%title(R1); %to add title

saveas(figure, title_name, 'pdf');

fprintf(1, 'Et voilà!');
